# Baricitinib therapy in critical COVID-19: plenty of promise, but no hard evidence yet

**DOI:** 10.1186/s13054-024-05191-9

**Published:** 2024-12-18

**Authors:** Seung-Hun You, Moon Seong Baek, Tae Wan Kim, Sun-Young Jung, Won-Young Kim

**Affiliations:** 1 Department of Global Innovative Drugs, The Graduate School of Chung-Ang University, Chung-Ang University, Seoul, Republic of Korea; 2 Division of Pulmonary and Critical Care Medicine, Department of Internal Medicine, Chung-Ang University Hospital, Chung-Ang University College of Medicine, Seoul, Republic of Korea; 3 College of Pharmacy, Chung-Ang University, Seoul, Republic of Korea

Dear Editor,

We would like to thank Wei et al. [1] for their interest in our recently published correspondence in *Critical Care* [2]. The authors share our enthusiasm for the comparison of baricitinib and tocilizumab therapies in patients with coronavirus disease 2019 (COVID-19) receiving mechanical ventilation (MV) and agree that the study findings are important. However, they raised several issues with respect to the methodology regarding confounding by indication.

The authors commented that the tocilizumab group had a higher severity of illness, which might have led to a bias in the outcome assessment of the baricitinib and tocilizumab groups, even after propensity score (PS) matching. Indeed, the tocilizumab group was more likely to exhibit higher Charlson Comorbidity Index and renal dysfunction, along with a greater frequency of renal replacement therapy than those of the baricitinib group (Table S1 in the paper) [2]. However, contrary to the authors’ concerns, the patients in the baricitinib group were more likely to receive neuromuscular blocking agents and extracorporeal membrane oxygenation. Hence, we respectfully disagree, at least in part, with their claim that tocilizumab was preferentially administered to patients with rapidly progressing or refractory conditions. In the Korean National Health Insurance Service database [3], it is not feasible to temporarily associate MV with drug administration (baricitinib or tocilizumab) during hospitalization due to the lack of timestamps. Hence, it was not possible to assess whether the duration of MV prior to drug administration was associated with the outcomes in our study.

We agree with the authors’ opinion that the study design was vulnerable to unmeasured confounders, although the groups were balanced with regard to the measured confounders using a robust model such as PS analysis. However, the current guidelines are based on the results of analyses that do not include direct comparison between the two drugs [4, 5]. Thus, observational studies are useful for providing data regarding the effectiveness of baricitinib and tocilizumab in patients with critical COVID-19. We also agree that the differences in treatment duration and pharmacodynamics may have resulted in a more favorable response to baricitinib. In fact, multiple oral administrations of baricitinib may potentially exhibit consistent drug concentrations, even in cases of gastrointestinal dysfunction commonly observed in critically ill patients [6].

Since the intolerance to enteral nutrition might reflect a more critical condition, we conducted a subgroup analysis of 30-day mortality according to total parenteral nutrition (TPN) therapy (yes or no) in response to the authors’ suggestion regarding the exclusion of patients who received TPN. TPN use was identified using the relevant procedure codes (aseptic preparation fee of parenteral nutrition [J0042] and/or nutrition support team consultation fee [AI600 and AI700]) [7]. A higher percentage of patients in the tocilizumab group received TPN than that in the baricitinib group (239/557 [42.9%] vs 188/557 [33.8%], respectively; standardized mean difference = 1.01). However, regardless of TPN use, patients who received baricitinib exhibited significantly lower mortality rates than of those who received tocilizumab (Fig. 1). Notably, patients who received TPN experienced lower mortality rates, thereby indicating that intravenous therapies may not be ideal surrogates of disease severity. This finding may also be attributed to the difficulties in providing active nutritional support during the COVID-19 pandemic [8].

**Fig. 1:**
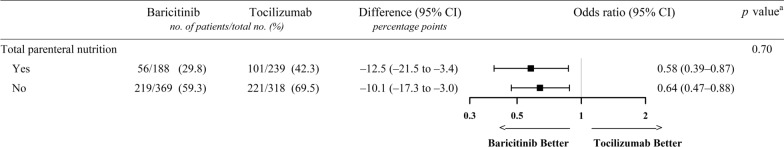
Association of baricitinib therapy on 30-day mortality according to total parenteral nutrition. Odds ratios (represented by squares) and 95% CIs (corresponding lines through them) were calculated for the propensity score-matched baricitinib (n = 557) and tocilizumab (n = 557) groups. ^a^ *p* values are for the interaction term. *CI* confidence interval

In conclusion, our study demonstrates that baricitinib may be a promising therapy for the treatment of patients with COVID-19 on MV. However, we agree with the authors’ observation that future studies would require more granular data, such as vital signs and laboratory values, to evaluate the association with baseline severity between the baricitinib and tocilizumab groups. Additionally, data on the timing of MV initiation and drug administration would be helpful in assessing the effects of early or late administration of baricitinib in patients requiring oxygen or MV. Finally, baricitinib or tocilizumab concentrations and inflammatory cytokine levels should be measured to enhance our understanding of the relationship between drugs and clinical response.

## Data Availability

All data generated or analyzed during this study are included in this published article.

## Abbreviations

COVID-19: Coronavirus disease 2019
MV: Mechanical ventilation
PS: Propensity score
TPN: Total parenteral nutrition
